# Job vacancy at *Eurosurveillance*

**DOI:** 10.2807/1560-7917.ES.2020.25.40.2010082

**Published:** 2020-10-08

**Authors:** Eurosurveillance editorial team

**Affiliations:** 1 European Centre for Disease Prevention and Control (ECDC), Stockholm, Sweden

The European Centre for Disease Prevention and Control (ECDC) invites applications for the following position:

 

**Scientific editor *Eurosurveillance***

The application deadline expires on 5 November 2020. 

 

For more information, please visit the ECDC website: 

https://ecdc.europa.eu/en/work-us/vacancies?f%5B0%5D=deadline_date%3A1

